# Looking Back From the Future: Perspective Taking in Virtual Reality Increases Future Self-Continuity

**DOI:** 10.3389/fpsyg.2021.664687

**Published:** 2021-06-09

**Authors:** Benjamin Ganschow, Liza Cornet, Sven Zebel, Jean-Louis van Gelder

**Affiliations:** ^1^Department of Social and Behavioral Sciences, Institute of Education and Child Studies, University of Leiden, Leiden, Netherlands; ^2^Department of Psychology of Conflict, Risk and Safety, University of Twente, Enschede, Netherlands; ^3^Department of Criminology, Max Planck Institute for the Study of Crime, Security, and Law, Freiburg im Breisgau, Germany

**Keywords:** virtual reality, perspective taking, future self-continuity, future self-vividness, two-chair dialogue, future self

## Abstract

In the current study, we tested a novel perspective-taking exercise aimed at increasing the connection participants felt toward their future self, i.e., future self-continuity. Participants role-played as their successful future self and answered questions about what it feels like to become their future and the path to get there. The exercise was also conducted in a virtual reality environment and *in vivo* to investigate the possible added value of the virtual environment with respect to improved focus, perspective-taking, and effectiveness for participants with less imagination. Results show that the perspective taking exercise in virtual reality substantially increased all four domains of future self-continuity, i.e., connectedness, similarity, vividness, and liking, while the *in vivo* equivalent increased only liking and vividness. Although connectedness and similarity were directionally, but not significantly different between the virtual and *in vivo* environments, neither the focus, perspective taking, or individual differences in imagination could explain this difference—which suggests a small, but non-significant, placebo effect of the virtual reality environment. However, lower baseline vividness in the *in vivo* group may explain this difference and suggests preliminary evidence for the dependency of connectedness and similarity domains upon baseline vividness. These findings show that the perspective taking exercise in a VR environment can reliably increase the future self-continuity domains.

## Introduction

Our future self is the beneficiary or unfortunate inheritor of all our major decisions and daily choices. During these choices, the tendency to act for the benefit of the future self has been shown to be contingent upon the degree of connection between the person's present and future selves, or future self-continuity (Hershfield, 2011; Hershfield and Bartels, 2018). These choices tend to accrue over time with future self-continuity positively correlating with better general well-being (Rutchick et al., 2018), improved mental health (McElwee and Haugh, 2010; Sokol and Eisenheim, 2016), better academic performance (Adelman et al., 2017), and greater personal net worth (Ersner-Hershfield et al., 2009). Importantly, an emerging body of evidence has shown that experimentally increasing future self-continuity in the present can also influence future-oriented behaviors contingent to these outcomes, such as increased exercise duration (Rutchick et al., 2018), promoting ethical decision making (Hershfield et al., 2012), reducing procrastination (Blouin-Hudon and Pychyl, 2015, 2017), and increasing savings behavior (Ersner-Hershfield et al., 2009; Bryan and Hershfield, 2012).

These changes in participants' behavior after these manipulations are not always strictly attributed to future self-continuity, but often studied through one of the three domains of future self-continuity proposed in Hershfield (2011), of similarity/connectedness, liking, and vividness. Future self- similarity/connectedness connotes an individual's belief in the stability of their “self over time” (Hershfield and Bartels, 2018), which broadly refers to any number of current beliefs, desires, or personality traits believed to remain similar between an individual's present and future selves (Bartels and Rips, 2010). Future self-liking is the positive or negative affect associated with the future self (Hoffner and Buchanan, 2005). Lastly, future self-vividness is the level of precision with which people can describe and imagine their future selves (Hershfield and Bartels, 2018). However, these domains of future self-continuity are rarely studied in concert and often assumed after manipulation. In the present study, we assess our manipulation over all the domains of future self-continuity.

Future-oriented behaviors are often influenced by relatively brief and subtle manipulations of the future self-continuity domains, such as through short writing assignments or embodying age-morphed avatars in virtual reality (VR) (Hershfield et al., 2018). To improve upon these promising manipulations, the current study tests a more rigorous and theory-driven manipulation that should improve each future self-continuity domain. Secondly, our study is novel to VR studies as we compare an *in vivo* or classic manipulation applied to an equivalent VR condition. All previous VR studies in embodiment literature compare VR to other VR conditions, which does not allow comparison to traditional methods or to measure the unintended added value of being in a VR environment. VR may impart an unintended effect similar to a sham condition in studies using transcranial magnetic stimulation or through other possible moderators explained later in the introduction. To the first end, we developed and tested a perspective taking exercise designed to increase the future self-continuity domains. To the second end, we also developed an equivalent VR environment to investigate the added value of a VR environment to an *in vivo* exercise.

Our manipulation expands upon prior experimental manipulations that seek to make participants, according to Hershfield, “aware that his or her future self is a living, breathing individual who is dependent on the choices of the current self”. (Hershfield et al., 2011, p. 33). These manipulations can create this awareness using two different modalities, one that requires the participant's active reflection and imagination to generate a future self or one that presents a visual representation of the participant's future self (Hershfield et al., 2018).

Reflective manipulations are often writing assignments and visualization exercises that prompt participants to imagine and reflect about who their future self will be in 10 or 20 years' time (Hershfield et al., 2012; Van Gelder et al., 2013; Rutchick et al., 2018; Sokol and Serper, 2019). Influencing scores through writing assignments have yielded small differences in future self-similarity, where improvements in similarity have not exceeded 0.5 (on a 7-point scale) in mean difference scores (Hershfield et al., 2012; Sokol and Serper, 2019, Study 1). Despite the modest difference scores, participants writing to their future self were 1.4 times more likely to work-out on a given day (Rutchick et al., 2018), reported more life satisfaction and more positive affect after an ego-deflating task (Sokol and Serper, 2019), were less likely to make delinquent choices in hypothetical scenarios (Van Gelder et al., 2013), and were less likely to condone unethical behaviors (Hershfield et al., 2012). Improving upon these relatively simple manipulations, a more intensive, 25-min visualization exercise reported in Sokol and Serper (2019, Study 2) resulted in a larger 0.8 increase (on a 7-point scale) in future self-similarity.

Presentational manipulations, in contrast, have participants view an age-morphed image or age-morphed avatar in VR and are similarly effective at influencing behaviors without requiring active reflection. For instance, Hershfield et al. (2011, Study 5) found that participants who viewed an age-morphed image of their future self reported a 1.1 increase in futures self-similarity (on a 7-point scale) between experimental and control groups and that this difference mediated the participants increased salary allocation toward a hypothetical retirement fund. Likewise, in Van Gelder et al. (2015), high-school students who received a message once a day from a social media account with their age-morphed profile picture reported 0.26 increase in future self-vividness, which mediated the reduction in self-reported delinquency during the study. Furthermore, interacting with age-morphed avatars in VR has been shown to increase savings behavior, reduce delay discounting (Hershfield et al., 2011, Study 1 and 2), and reduce cheating behavior (Van Gelder et al., 2013).

To sum up, manipulations that improve future self-continuity domains can influence future-oriented behaviors and yet are relatively undemanding and focus on improving only one future self-continuity domain. Therefore, a more rigorous and theory-driven technique may improve all of the domains and possibly to a greater degree. Our study tests a novel perspective taking exercise specifically designed to evoke vivid mental imagery of a positive future self and reflection about the present self to increase the future self-continuity domains.

The exercise is based on the two-chair technique—a perspective switching method successfully utilized in a wide-range of therapies (Wagner-Moore, 2004; Kellogg, 2014; Pugh, 2017). The technique aims to help clients emotionally engage with and cognitively reflect on “unfinished business” or thoughts, feelings and memories which they are unable or unwilling to examine from their own perspective (Greenberg and Malcolm, 2002). By taking the perspective of a “significant other,” or someone central to the unfinished business, the client can feel, think, and act in ways consistent with how this other would feel, think, and behave (Pugh, 2017). Typically, the two-chair technique has the client sit opposite a vacant chair in which he or she imagines the significant other. The client asks the (imaginary) significant other a question, then proceeds to take the seat of the significant other and answers the question role-playing as the significant other. With the alternative perspective thus engaged, the therapist directs the dialogue toward both emotional engagement and cognitive reflection with the “unfinished business” (Kellogg, 2014, p. 19).

We adapted this technique, which we call the perspective taking exercise, to help participants to engage as their successful future self emotionally and cognitively. The exercise follows the same process as a typical two-chair technique, except the significant other they take the perspective of is their “future self in 10 years” and the unfinished business is replaced with a “great accomplishment” that their future self has achieved. The exercise is self-guided whereby the participant, from the perspective of their future self, answers a sequence of questions designed to help the participant imagine what it would feel to be and reflect on how to become their successful future self.

We based the questions on previous mental contrasting interventions, where participants are first asked imagine a positive future goal (e.g., receiving a high mark on an exam) and secondly to contrast this positive future with likely obstacles in the present (e.g., playing video games instead of studying) (Oettingen and Gollwitzer, 2001; Duckworth et al., 2013). Previous mental contrasting interventions using this sequence have resulted in positive outcomes to future-oriented behaviors, including school performance, dieting, and exercise behavior (Oettingen, 2012). The questions in our perspective taking exercise follows a similar process to mental contrasting: first the elaboration of a positive future self followed by questions contrasting the present self to the positive future self.

The perspective taking exercise may increase future self-similarity and connectedness as participants contrast their current and future selves, while future self-vividness and liking because participants describe an accomplished, autonomously chosen future self. However, the effectiveness of the exercise may be limited by the willingness of the participants to take the role of their future self, which may be improved upon through implementation in a standardized VR environment.

The empty chair technique is generally implemented in the controlled environment of the therapist's office with the encouragement of a therapist to role-play. Ideally, the perspective taking exercise can be implemented anywhere with little to no supervision, but we would expect that this exercise would be rendered ineffective for certain individuals who lack focus, find the routine of the exercise particularly odd, or lack imagination. Consequently, replacing the laboratory room with a virtual environment may help reduce these disadvantages.

Focus may be improved by the VR environment because it limits the possible of distractions through the restriction of actions and movement in comparison to a laboratory environment. That is, in laboratory or in natural settings the pertinent stimuli may be controlled but the surrounding environment is not, while in VR, the participant responds to stimuli while immersed in a larger, controlled virtual environment (Wilson and Soranzo, 2015).

The perspective taking exercise *in vivo* has another drawback—the performance of the exercise can feel unnatural. Participants talk to an empty chair, imagine their future self in that chair, and are required to physically switch seats while mentally switching roles. The oddity of the exercise may reduce the compliance of the two-chair routine with some participants and lessen the effectiveness of the exercise. Therefore, putting participants in an “unreal” affordance-free environment may enhance the perspective taking of participants who would be uncomfortable with performing the routine of the two-chair exercise in the laboratory. Similarly, Jouriles et al. (2009, 2011) had participants role-play as potential victims of sexual assault in a resistance training in an *in vivo* condition and an equivalent VR environment. They found that the VR condition was more effective at eliciting participant emotion and immersion in the role-play and speculate that VR may have made participants less self-conscious in a virtual environment than in the laboratory. Therefore, we think the VR environment may increase perspective taking.

VR may also benefit participants with low imaginative ability. Blouin-Hudon and Pychyl (2015) reported imaginative ability to be correlated with a composite score of future self-similarity and connectedness (*r* > 0.2), suggesting that high imagination may help in envisioning future selves. In VR, the visual environment and chair switching are generated for the participant, freeing up cognitive resources recruited for the exercise. This suggests that participants low in imaginative ability may benefit more from the perspective taking exercise in VR environment in comparison to *in vivo*.

In summary, the aim of the current study is two-fold, to test the efficacy of the perspective taking exercise and examine possible differences between the VR and *in vivo* conditions.

The efficacy of the perspective taking exercise is investigated through means-level difference scores between baseline and post-experiment future self-continuity domains in comparison to an inert control condition. In addition, efficacy is analyzed at the subject-level, to investigate how baseline individual differences in the future self-continuity domains may additionally be influenced by the condition of the exercise, i.e., the effect from the exercise may be stronger for those with little relationship to their future self in comparison to those who are already familiar with their future self. This is investigated using a controlled pretest-posttest experimental design to detect two-way interactions between baseline values of future self-continuity domains and experimental conditions.

To the second aim, we examined how the possible added value of only the VR environment compared to the *in vivo* environment as the VR environment may improve focus, increase perspective taking and may be more effective for participants with low-imaginative ability.

## Materials and Methods

### Sample

Participants were university undergraduate students recruited through word of mouth and from the faculty's participant recruitment pool and were remunerated with course credit or a €6 gift voucher. A power analysis was conducted to estimate the sample size for a one-sided *t*-test between baseline to post-experiment future self-similarity, liking and vividness. The analysis estimated 26 participants per group based upon an effect size of *Cohen's d* = 0.5, with 0.80 power, and 0.05 alpha. The effect size was based upon effect sizes reported in studies manipulating future self-continuity (*Cohen's d Range* = *0.36–*0.77; Bartels and Urminsky, 2011; Hershfield et al., 2011, 2012; Sokol and Serper, 2019). We recruited a final sample of 93 participants (*M*_age_ = 21.7, *SD* = 2.28, *Range* = 18–32; 70% female). The study was approved by the BMS Ethics Committee of the University of Twente (#180047).

### Materials

#### Baseline Questionnaire

Future self-similarity was assessed with two items following Ersner-Hershfield et al. (2009), respectively “How connected do you feel to yourself 10 years into the future?” and “How similar do you feel to yourself 10 years into the future?” Both items were rated on a visual scale of seven pairs of increasingly overlapping circles representing their present and future self. Test-retest reliability of controls from baseline to post-experiment was modest for connectedness [*r*(30) = 0.58, *p* < 0.001] and satisfactory for similarity [*r*(30) = 0.82, *p* < 0.001]. We did not combine these domains as there is mixed use in the literature of these items, sometimes they are combined (e.g., Adelman et al., 2017), only use connected (e.g., Bartels and Urminsky, 2011), or only similarity (e.g., Ersner-Hershfield et al., 2009). Future-self liking was measured by the item: “Tell us how you feel about your future self 10 years from now” on a 7-point scale *(don't like at all-like a lot*). Test-retest reliability was modest [*r*(30) = 0.58, *p* < 0.001]. Future self-vividness was adopted from the 6-item scale by Van Gelder et al. (2015). Due to time constraints, participants were asked two of the vividness items, “I find it easy to imagine myself 10 years from now” and “I find it easy to describe myself 10 years from now” and indicated their agreement on a 7-point scale ranging from (*strongly disagree to strongly agree*; all following agreement scales use the same scale intervals). Correlations between the two measures were high at both time points [*r*(30) = 0.84, *p* < 0.001] and test-retest reliability of the composite scale was satisfactory [*r*(30) = 0.80, *p* < 0.001].

Imaginative ability was measured using the Vividness of Visual Imagery Scale (VVIQ) from Marks (1973). Participants imagine a series of four scenes (e.g., “Visualize a rising sun.”) with four vignettes to each scene (e.g., “The sun rising above the horizon into a hazy sky,” “The sky clears and surrounds the sun with blueness.”). Participants rate how vividly they imagined each vignette on five statements, “No image at all, you only “know” what you are thinking about.” to “Perfectly clear and vivid as real seeing.” Scores were summed, which had acceptable reliability (α = 0.82).

Lastly, participants completed an unconstrained thinking exercise where they wrote for 5 minutes about an accomplishment they wanted to achieve in the next 10 years. The prompt was based on the “best possible selves” writing assignment (King, 2001). Participants later used these answers basis for their successful future self that the participant would role-play in the perspective taking exercise (see Supplementary Material).

#### Perspective Taking Exercise

**Figure 1:**
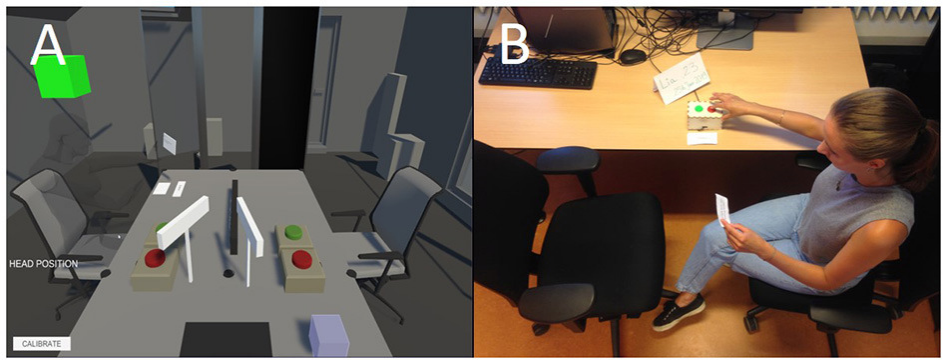
Bird's-Eye view of the perspective taking exercise in VR and *in vivo*. Participants VR condition (Panel **A**) and *in vivo* (Panel **B**).

The perspective taking exercise begins with the participant setting a goal in 10-years' time, followed by a tutorial of the two-chair technique, and completion of the two-chair technique. To set the goal, participants were asked to remember what they wrote about in the unconstrained thinking exercise and choose one achievement that they would accomplish in around 10 years. Subsequently, the *in vivo* participants sat across from an empty chair in the laboratory and the VR participants sat across from an empty chair in a VR environment. In both conditions, participants saw a card displaying the name, age, and date of their present self on one side of the table, and the name, age, and date of their future self on the other side of the table. Next to the name card was a stack of questions and a box with a red “record” and a green “play” button (see Figure 1). Both conditions were similar, but in VR pushing the green button also switched the participant to the empty chair opposite with an added auditory “whoosh” and visual “fade-out to fade-in” during the change, while *in vivo* participants switched chairs under their own motility. This procedure is inspired by Slater et al. (2019) where participants performed a VR self-therapy two-chair dialogue that had participants switching between their current self and Sigmund Freud.

**Figure 2:**
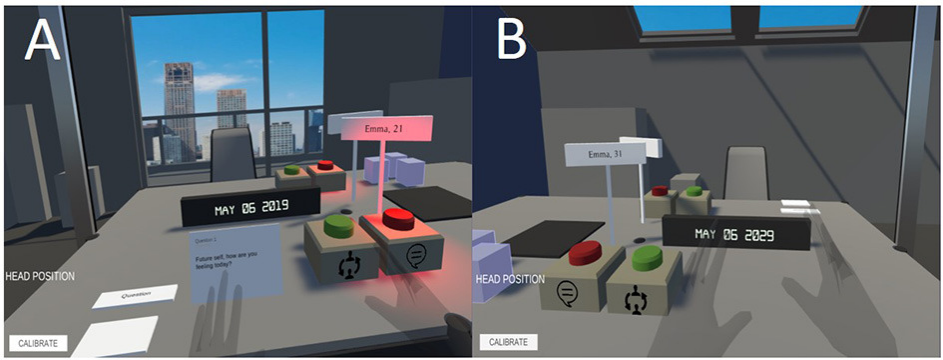
The process of the perspective taking exercise. Participants in the present self **(A)** pick up a question card and press the red button to record the question. Participants then press the green button to switch chairs to the future self perspective **(B)**. As their future self, they press the red button to record their answer. Participants then press the green button to switch chairs to the perspective of their present self **(A)** and the recorded answer plays. Once the recording is finished, the participant picks up the next question card until all nine are finished. The process is the same in VR and *in vivo* conditions.

The routine of the perspective taking exercise was first practiced in a tutorial identical to the exercise itself. Participants picked up a tutorial question card, started recording, and asked the question written on the card to their future self. After, they stood up (or in VR, pressed the green button) and sat in the chair of their future self. Then, from the perspective of their future self, they listened to the previous question and recorded an answer to the present self's question. Lastly, participants returned to sitting in the present self chair and listened to the recorded answer from their future self (see Figure 2). In addition to the tutorial, participants in the VR condition performed an avatar embodiment exercise where they faced a mirror and raised their hands, shook their head, and moved their torso (Hershfield et al., 2011; Van Gelder et al., 2013). When participants (in both conditions) performed one error-free question and answer, the researcher left the room. The participant completed the nine questions following the same process (see Supplementary Material). After the exercise, participants were asked about their experience and completed the post-experiment questionnaire.

#### Post-Experiment Questionnaire

In the post-experiment questionnaire, all participants completed the VVIQ and future-self continuity domain measurements (see Baseline Questionnaire). Participants in the *in vivo* and VR conditions additionally completed the user engagement scale and the perspective taking index.

The User Engagement Scale–Short Form (O'Brien et al., 2018) measures user engagement with the perspective taking exercise through nine questions on the 7-point agreement scale. We calculated a total engagement score (α = 0.86) composed of the three subscales. These are “focused attention” (α = 0.72), indicating how cognitively absorbing the exercise was, “perceived utility” (α = 0.72), indicating how the task elicited confusion and frustration, and “reward” (α = 0.92) indicating how worthwhile, interesting, and rewarding the exercise was. The “focused-attention” subscale was used as our measure of focus.

Perspective taking is measured in two ways, an embodied perspective, where participants “feel” like their future self and a cognitive perspective, where participants begin thinking differently than normal when answering questions as their future self. Cognitive perspective taking was assessed through three statements on the 7-point agreement scale including the following items: “When sitting in the chair of my future self, my thoughts changed from how I would normally think,” “I was surprised by the answers I came up with,” and “I gave different answers than I normally would have.” The measurements had acceptable reliability (α = 0.77), so a composite perspective taking score of these measurements was calculated.

Embodied perspective taking was assessed through two questions of the 7-point agreement scale adapted for non-VR exercises (Slater et al., 2010). Included were: “When sitting in the chair of my future self, I felt as if I was my future self” and the reverse coded “When sitting in the chair of my future self, I felt as if I was someone else.” The reliability of the composite was low (α = 0.50), and the results were therefore analyzed as single items.

### Procedure

#### One Week Before the Experiment

Following randomization, participants completed the baseline questionnaire online. Participants in the control condition did not write the “unconstrained goal assignment.” After completion, to reduce recency bias, participants reserved a date at least one-week later to come to the laboratory to take part in a “future self visualization exercise.”

#### Experiment

Participants in the *in vivo* and VR condition were given a general explanation of the perspective taking exercise, informed of their anonymity, and gave consent. After consent, control participants completed the baseline questionnaire for the second time and could choose to go home or complete the perspective taking exercise. Participants in the *in vivo* and VR conditions completed the perspective taking exercise and the post-experiment questionnaire.

## Results

To determine whether the perspective taking exercise was effective and in which condition, we first compared difference scores between baseline and post-experiment future self-continuity domains (also referred to as “domain scores”) of connectedness (FSC), similarity (FSS), liking (FSL) and vividness (FSV). We then examined the interaction of baseline domain scores by condition, while controlling for placebo effects and regression toward the mean. Then, we investigated how participants experienced the perspective taking exercise by analyzing mean scores of focus, perspective taking, and examined imaginative ability as a potential moderator. Lastly, we included *post-hoc* correlations of baseline and post-exercise domain scores between conditions to identify inconsistencies between conditions.

### Perspective Taking Exercise

To assess whether the perspective taking exercise increased domain scores, we first examined mean difference scores through paired sample *t*-tests between baseline and post-experiment measurements across conditions. Mean baseline domain scores were similar between conditions in FSC, FSS and FSL, although there is a noticeable 0.8 difference in mean FSV favoring VR to *in vivo*. Only significant mean difference scores are reported here (for all difference scores, see Table 1).

**Table 1:** Means, standard deviations, and difference scores between baseline and post-experiment of the domains of future self-continuity.

			**Baseline**		**Post-experiment**		**Difference**	
	**Condition**	**N**	**Mean**	**SD**	**Mean**	**SD**	**Mean**	**SD**
Connected	Control	32	3.53	1.85	3.78	1.64	0.25	1.61
	*in vivo*	30	3.57	1.79	4.13	1.43	0.57	1.77
	VR	31	3.84	1.39	4.90	1.49	1.06^***^	1.31
Similarity	Control		3.31	1.35	3.09	1.28	−0.22	0.8
	*in vivo*		3.23	1.43	3.4	1.35	0.17	1.44
	VR		3.58	1.36	4.13	1.41	0.55^*^	1.34
Liking	Control		5.72	1.17	5.72	0.68	0	0.95
	*in vivo*		5.5	1.04	6.23	1.1	0.73^***^	1.01
	VR		5.84	1.16	6.32	0.6	0.48^*^	1.12
Vividness	Control		3.77	1.72	3.94	1.6	0.17	1.07
	*in vivo*		3.43	1.63	4.62	1.27	1.18^***^	1.61
	VR		4.23	1.48	5.02	1.24	0.79^***^	0.98

*Asterisks indicate significance levels at*

* *p < 0.05;*

** *p < 0.01;*

*** *p < 0.001*.

Participants in the VR condition showed significant medium to large increases in FSC [*M* = 1.1, *t*(30) = 4.51, *p* < 0.001, 95% CI (0.58, 1.55)], FSS [*M* = 0.5, *t*(30) = 2.28, *p* = 0.029, 95% CI (0.06, 1.04)], FSV [*M* = 0.8, *t*(30) = 4.48, *p* < 0.001, 95% CI (0.43, 1.15)] and FSL [*M* = 0.5, *t*(30) = 2.40, *p* = 0.02, 95% CI (0.07, 0.90)]. Participants in the *in vivo* condition, showed medium to large significant differences in FSL [*M* = 0.7, *t*(29) = 3.96, *p* < 0.001, 95% CI (0.35, 1.11)] and FSV [*M* = 1.2, *t*(29) = 4.04, *p* < 0.001, 95% CI (0.58, 1.78)], but not in FSC [*M* = 0.6, *t*(29) = 1.75, *p* = 0.09, 95% CI (−0.10, 1.23)] and FSS [*M* = 0.2, *t*(29) = 0.63, *p* = 0.53, 95% CI (−0.37, 0.70)].

*T*-tests on difference scores between VR and *in vivo* conditions yielded no significant differences across the four domains. While in the control condition, as expected, no differences emerged in any of the measures (*M* < 0.25).

In sum, the perspective taking exercise in VR was effective at increasing all domain scores, whereas the *in vivo* condition only increased FSL and FSV, but not their FSC or FSS.

### Estimating Condition by Baseline Interactions While Controlling for Regression and Placebo Effects

To estimate possible interactions between baseline scores and conditions, the effects of the perspective taking exercise, we used the differential response to treatment model from Weigel and Narvaez (1991). It is an ANCOVA model that estimates the main effect of the exercise and the interaction of baseline future self-continuity domain scores with conditions. The model also accounts for contamination of these estimations from regression to the mean over repeated measurements (e.g., Dawes and Mulford, 1996; Linden, 2013) and a placebo effect on the day of the experiment (Boot et al., 2013). In our study, we account for regression to the mean between measurements because participants may be unfamiliar to the concept of the future self or confused by the unique format of the overlapping circles question. We further account for between condition placebo effects on the day of the experiment because previous VR condition participants may have relayed their VR experience to participants in the non-VR conditions, raising their expectations and may bias questionnaire responses. This model is identified as:

$$\begin{array}{l} \Delta \left(Y-X_{2}\right)=\beta_{0}+\beta_{1}X_{1}+\beta_{2}\left(X_{2}-\bar{X_{2}}\right)+\beta_{3}X_{1}\left(X_{2}-\bar{X_{2}}\right)+\epsilon \end{array}$$

where:

$$\begin{array}{l} \Delta \left(Y-X_{2}\right)=difference\;score\;between\;baseline\;and\;post\\ \;-experiment\;component\;scores\\ \;X_{1}=condition\\ \;X_{2}=participant\;baseline\;score\\ \;\bar{X_{2}}=grand\;mean\;of\;baseline\;scores\\ \;\epsilon =error\;term \end{array}$$

The future self-continuity domain difference Δ(*Y* − *X*_2_) and baseline $\left(X_{2}-\bar{X_{2}}\right)$ scores are mean centered to correctly estimate interpretable interaction effects with normally distributed data (Weigel and Narvaez, 1991).

The β_0_ intercept estimates what is shared commonly by the three conditions that is independent of the baseline. It is interpreted as a placebo effect from being in the control condition. β_1_ estimates the experimental effect due to completing the exercise in VR or *in vivo*. $\beta_{2}\left(X_{2}-\bar{X_{2}}\right)$ estimates regression toward the mean, or the effect that is common to all conditions and dependent on baseline. $\beta_{3}X_{1}\left(X_{2}-\bar{X_{2}}\right)$ is the condition by baseline interaction effect, which estimates the effect that is unique to the *in vivo* and VR conditions, but dependent upon the participant's baseline domain score. If β_3_ < 0, the exercise would have a greater effect for participants with a baseline domain score below the sample mean, while the opposite for those above the sample mean.

Data was analyzed using RStudio (Dayal, 2015) running R (R Core Team, 2017). Final models were selected by stepwise change in adjusted *R*^2^ beginning with the null model (β_0_ + β_2_), adding β_1_, and then adding interactions. Gender and age were controlled for in each model but were not strong or significant predictors to be included in final models. The interaction models explained significantly more adjusted *R*^2^ and were retained (data and code available upon request). Model residuals were normally distributed for FSC, FSS and FSV models. FSL model residuals were non-normally distributed and the baseline and, consistent with FSL reported in Hershfield (2011), the post-experiment FSL scores were negatively skewed (Shapiro-Wilk's *W* = 0.88, *p* < 0.001), indicating a ceiling effect. FSL estimations may be unreliable.

In-line with the findings from the paired sample *t*-tests, while controlling for placebo effects and regression toward the mean, model estimates show that the perspective taking exercise in VR significantly improved the difference scores in FSC, FSS, and FSV, while having a smaller, significant effect on FSL. VR participants reported no meaningful baseline by condition interactions, which indicates that the change in domain scores was largely similar for all participants in VR. Main effect estimates for the *in vivo* condition indicate that, compared to the control condition, the *in vivo* exercise significantly improved FSV and FSL, but had no effect on FSC and FSS. Additionally, the negative interaction between the perspective taking exercise on baseline FSV scores estimates that the *in vivo* exercise was more effective for participants with low-baseline FSV scores (see Table 2). The interaction estimates that one point below the sample mean improves the exercise effect by 50%, while likewise reducing for a point above. However, given that there is a 0.8 baseline imbalance of FSV between participants in the VR and *in vivo* condition, we cannot conclude that the *in vivo* condition is more effective with low-baseline FSV participants. The baseline imbalance may also suggest that participants with low-baseline FSV tend to respond better to the exercise.

**Table 2:** Regression coefficients and 95% confidence intervals of control, *in vivo*, VR, and baseline interactions on difference scores of the domains of future self-continuity.

	**Connectedness**	**Similarity**	**Liking**	**Vividness**
β_0_ Control (Placebo)	0.19	−0.23	0.02	0.16
	(−0.27, 0.64)	(−0.60, 0.14)	(−0.22, 0.26)	(−0.19, 0.51)
β_2_ Baseline^*^Control (Regression to the mean)	−0.48^***^	−0.225	−0.66^***^	−0.26^**^
	(−0.73, −0.24)	(−0.50, 0.05)	(−0.87, −0.45)	(−0.47, −0.06)
β_1_ *in vivo*	0.32	0.32	0.63^***^	0.77^***^
	(−0.34, 0.97)	(−0.21, 0.85)	(0.28, 0.99)	(0.26, 1.27)
β_1_ VR	0.94^***^	0.87^**^	0.58^***^	0.78^***^
	(0.293, 1.55)	(0.338, 1.41)	(0.22, 0.94)	(0.28, 1.29)
β_3_ Baseline^*^in vivo	−0.19	−0.33	0.25	−0.42^***^
	(−0.55, 0.18)	(−0.72, 0.06)	(−0.08, 0.57)	(−0.72, −0.11)
β_3_ Baseline^*^VR	0.111	−0.22	−0.18	−0.1
	(−0.31, 0.52)	(−0.62, 0.16)	(−0.48, 0.13)	(−0.42, 0.21)
Observations	93	93	93	93
*R*^2^	0.36	0.29	0.58	0.43
Adjusted *R*^2^	0.32	0.25	0.56	0.4
Residual std. error (df = 86)	1.31	1.11	0.71	1.0
F statistic (df = 5; 86)	10.02^***^	7.22^***^	24.1	13.31^***^

*Asterisks indicate significance levels at*

* *p < 0.05;*

** *p < 0.01;*

*** *p < 0.001*.

Regression toward the mean (β_2_ Baseline^*^Control) was significant and moderately large with FSC and FSL, while weakly influencing FSV. This highlights the substantial influence of measurement error between baseline and post-experiment measurements. However, there were no significant placebo effects observed between the measurements on the baseline and post-experiment in the control condition (β_0_ in the model), which rules out expectancy bias from being in a non-VR condition and other unintended between condition effects.

### Relationship of Focus, Perspective Taking, and Imaginative Ability Between VR and *in vivo*

Correlations between the engagement subscales, imaginative ability and perspective taking were performed between the FSC, FSS, FSL, and FSV difference scores. No significant correlations were found, which indicated that the proposed explanatory variables were not related to the difference scores (see Table 3).

**Table 3:** Correlations between of engagement subscales, embodiment, imaginative ability, and the difference scores from baseline to post-experiment in domains of future self-continuity.

	**Connectedness**	**Similarity**	**Liking**	**Vividness**
Focus	0.05	0.16	0.19	−0.01
Reward	−0.14	−0.13	0.07	−0.1
Perceived utility	−0.13	0.13	−0.1	−0.1
Engagement score	−0.09	0.16	0.03	−0.13
VVIQ	0.14	0.04	0.02	0
Cognitive perspective taking	−0.06	0.1	0.11	0.01
Embodied perspective taking 1	0.13	0.22	0.18	0.03
Embodied perspective taking 2	0	−0.1	0.26	−0.12

*Perceived utility is a measure of frustration and confusion while taking part in the perspective taking exercise. VVIQ is the vividness of visual imagery questionnaire, measure of imaginative ability. Embodied Perspective Taking 1 was “felt like I was my future self” and 2 was the reverse coded “I felt as if I was someone else.”*

We measured focus through the focused attention subscale of the User Engagement Scale—Short Form. Focus was not significantly different between conditions; however, the direction favored the VR condition [VR: *M* = 5, *SD* = 1.6; *in vivo*: *M* = 4.5, *SD* = 1.3, *t*(58.1) = 1.41, *p* = 0.17]. Additionally, no meaningful differences were reported between conditions for the reward and perceived utility subscales.

Cognitive perspective taking was examined across conditions with respect to the degree to which participants in the perspective taking exercise felt they thought differently while in the perspective of their future self. Mean scores were significantly higher in the *in vivo* condition (*M* = 4.7, *SD* = 1.8) than in the VR condition [*M* = 4.1, *SD* = 1.5; *t*(58.9) = −1.98, *p* = 0.05]. Embodied perspective taking, or how the participants felt they had become their future self, was not different for either of the two questions (see Supplementary Material).

Finally, we examined our expectation that participants with low imagination, measured by the Vividness of Visual Imagery Scale (VVIQ), would have higher domain scores in the VR condition. Controlling for baseline domain scores, a regression of the domain scores by both VVIQ scores and interaction of VVIQ by condition revealed no significant relationships. Thus, contrary to expectations, we neither replicated the correlation between VVIQ and FSS reported in Blouin-Hudon and Pychyl (2015) nor found the VR environment to be more effective for participants with low imagination.

### *Post-hoc* Correlation Analysis Between Baseline and Post-Experiment Domain Scores in VR and *in vivo*

The previous analyses demonstrate that VR had significant differences in all domain scores in comparison to the controls and non-significant but directional differences between VR and *in vivo*. Since we cannot provide strong answers as to whether the possible added value of VR occurred from focus, perspective taking or imaginative ability, we explored the correlations between baseline domain scores and post-experiment domain scores in both the VR and *in vivo* conditions to investigate the relative strengths of each exercise.

Results show that the pattern of correlations between baseline and post-experiment remains similar between conditions. Correlations between baseline and post-experiment FSC and FSS scores remained consistently similar between conditions. However, in the VR condition, FSV was correlated strongly with post-experiment FSC [*r*(30) = *0.5*9, 95% CI (0.30, 0.78)], FSS [*r*(30) = 0.54, 95% CI (0.23, 0.75)], FSL [*r*(30) = 0.50, 95% CI (0.18, 0.72)], and FSV [*r*(30) = 0.75, (0.54, 0.87)]. This pattern of results was different from the *in vivo* condition with small correlations for post-experiment FSC [*r*(31) = 0.09, 95% CI (−0.28, 0.44)] and FSS [*r*(31) = 0.19, 95% CI (−0.18, 0.52)] and large correlations for both FSL [*r*(31) = 0.40, 95% CI (0.05, 0.67)] and FSV [*r*(31) = 0.41, 95% CI (0.06, 0.67)] (see Supplementary Material).

## Discussion

The purpose of this study was to investigate whether looking back from the perspective of one's future self would strengthen the similarity/connectedness, liking, and vividness domains of future self-continuity. Using a randomized pretest-posttest design, we tested the efficacy of the perspective taking exercise and examine possible differences between a VR and *in vivo* environment. We examined how the possible added value of only the VR environment compared to the *in vivo* environment as the VR environment may improve focus, increase perspective taking, and may be more effective for participants with low-imaginative ability.

This study yielded two key findings: (1) the *in vivo* condition was only effective at improving liking and vividness in comparison to controls; whereas (2) in the VR condition, all domains of future self-continuity can be substantially improved with our perspective taking exercise. However, we found no explanation for the difference between the VR and *in vivo* environments due to focus, perspective taking, embodiment or individual differences in imaginative ability. We speculate that this difference may either be due to an unmeasured feature of VR or alternatively, a lower baseline vividness scores in the *in vivo* sample. This suggests an important role of future self-vividness in improving connectedness and similarity.

Our 25-min VR exercise reliably increased all domains of future self-continuity by 0.8, which is larger than previous studies using writing assignments and the first exercise designed to improve all domains (Bartels and Rips, 2010; Hershfield et al., 2012; Sokol and Serper, 2019).

Our exercise demonstrated increased effects on future self-similarity, similar to a guided interview implemented in Sokol and Serper (2019). The interview lasted 25 min which included visualizing the past and future, creating a life-history narrative, and reflecting upon past and future behaviors. The similarities between the content, duration, and the 0.7–0.8 difference to similarity of both exercises suggest that more intensive exercises may improve the future self-continuity domains better than brief manipulations. However, a clear advantage of our perspective-taking exercise over the Sokol and Serper interview is that it can be completed alone or with little to no supervision. This is especially advantageous in combination with VR, as this allows for researchers to study the future self remotely and in a controlled virtual environment. Additionally, the questions of the perspective taking exercise can be modified toward different interventions, such as adding implementation intentions to a goal setting exercise (Duckworth et al., 2013). In sum, the VR exercise is useful when a larger improvement to domains of future self-continuity is desired, to improve multiple domains, and designed for remote use.

However, our study cannot explain why the *in vivo* exercise was non-significantly but directionally less effective at increasing connectedness or similarity in comparison to the VR condition. For all explanatory variables, there were only small differences (>0.4) between the VR and *in vivo* conditions, with the exception an increase in cognitive perspective taking in the *in vivo* condition. Even so, the explanatory variables themselves were not correlated with the difference scores of the future self-continuity domains, rendering them irrelevant to the efficacy of the perspective taking exercise itself. We also examined *post-hoc* differences between the two conditions for reward and perceived utility (a measure of frustration with the task) user-engagement subscales and again found no mean-level differences or correlations with the future self-continuity domains. Moreover, we controlled for gender and age and the interaction between age and condition in each of our models, but all explained little meaningful variance. We therefore conclude that the explanatory variables measured, such as focus or cognitive perspective taking, could not capture how the perspective taking exercise improved the future self-continuity domains. Also, these variables could not distinguish why the VR condition provided marginally better, but non-significant, differences in similarity and connectedness. This lack of evidence may present a possible placebo effect of the VR environment.

Alternatively, the lack of effect for connectedness and similarity in the *in vivo* condition may arise from having a sample with unusually low future self-vividness (almost 0.8 lower), which implies that improving vividness may be necessary for improving similarity and connectedness. Further, our regression models found a baseline vividness by *in vivo* interaction that only occurs in the *in vivo* subgroup with low vividness. This is similar to the finding from the longitudinal study in Blouin-Hudon and Pychyl (2017), which reported that higher baseline future self-vividness was related to greater improvement in future self-similarity. This evidence leads us to speculate that our participants may have found it difficult to feel connected and similar to their future self if they could not initially describe or imagine their future in sufficient detail, independent of the exercise. To explore this possibility, we examined the correlations from baseline to post-exercise future self-continuity domains between VR and *in vivo*. If participants with low vividness found it more difficult to feel more connected and similar their future selves, we would expect to find different relationships between conditions from baseline to post-exercise similarity and connectedness. As expected, we found the correlations between baseline vividness and all post-exercise domain scores were large and significant in VR (*r* > 0.5), whereas *in vivo* baseline vividness scores were only related to post-exercise liking and vividness domains and not significantly correlated with connectedness and similarity. Future studies should examine the possibility of high baseline vividness being a pre-requisite for greater improvement to similarity and connectedness.

### Strengths and Limitations

One strength of our experimental design is that it allowed us to account for regression to the mean, which had a surprisingly large influence on the estimations of future self-connectedness, while also with liking and vividness. Regression to the mean often indicates measurement error between measurements (Linden, 2013; Twisk et al., 2018), which in our study may signal that the questions about connectedness, liking, and vividness were difficult to understand for participants. When using these measures, measurement error should be mitigated by accounting for regression to the mean, including more items in the questionnaire, or through better explanation to participants of what is meant by connectedness, liking and vividness.

Likewise, it is also important to recognize that we use a healthy university student sample, a population which likely has higher baseline future self-liking scores. This may be because young adults have been shown to overestimate future life satisfaction in comparison to older adults (Löckenhoff and Rutt, 2017). Future research should examine age-related disparities in future self-liking may also exist in samples with different socio-demographic backgrounds and also if disparities exist in other future self-continuity domains.

Lastly, our exercise was not designed to leverage the proven results of representational future self-avatars in VR, but to examine the added value of the VR environment when performing the perspective taking exercise. Studies have shown that controlling the avatar of one's age-morphed future self in VR decreased cheating behavior (Van Gelder et al., 2013) and increased retirement allocation from a hypothetical salary (Hershfield et al., 2011). Realistic age-morphed images and avatars may also improve vividness scores by overcoming “failures in imagination” as they viscerally present to participants a likely and believable future self (Loewenstein, 1996; Hershfield and Bartels, 2018). Future studies should investigate whether controlling an age-morphed virtual representation in the VR perspective taking exercise can further improve the effect, especially over and above the effect due to the “sham” VR condition in the current study.

A further limitation is that empty chair exercises generally take place within a therapist's office, in contrast, our laboratory was set-up to mimic the office within the virtual environment. The VR environment, therefore, may have been perceived as more inviting or enjoyable than the laboratory environment. However, this would likely have positively influenced participants' perspective taking and user-engagement scores in the VR condition, which we found no evidence for.

### Conclusion

This study investigated how more cognitively demanding exercises can help participants explore and reflect upon who they want to become. The perspective taking exercise asks participants to look back from the future and provide guidance to their present self, which helped the participants improve their ability to imagine and like their future self. Additionally, participants in the VR environment consistently improved their similarity and connectedness toward their future self. It is not yet clear why only the VR environment was marginally better than *in vivo*—but evidence suggests an important role for vividness as a catalyst for connectedness or that VR may have an added placebo effect when performing this exercise. In addition VR environment is also standardized, can be applied remotely, and future studies should improve this exercise with age-morphed avatars.

## Data Availability Statement

The raw data supporting the conclusions of this article will be made available by the authors, without undue reservation.

## Ethics Statement

The studies involving human participants were reviewed and approved by Behavioral and Management Sciences. The patients/participants provided their written informed consent to participate in this study. Consent was obtained from the relevant individuals the publication of any potentially identifiable images or data included in this article.

## Author Contributions

BG carried out the experiment and wrote the article. All authors helped with designing the experiment, data analyses, and revision.

## Conflict of Interest

The authors declare that the research was conducted in the absence of any commercial or financial relationships that could be construed as a potential conflict of interest.
