# Supplementary material for: Looking Back From the Future: Perspective Taking in Virtual Reality Increases Future Self-Continuity
Source: Front Psychol. 2021 Jun 9;12:664687. doi: 10.3389/fpsyg.2021.664687 (PMC8219936; doi:10.3389/fpsyg.2021.664687)
Supplement: Supplementary file 1 [file Table_1.docx]

Supplementary Material

# Unconstrained Writing Exercise

“Please try to imagine that in the next 10 years, you can achieve whatever you want.

There are no limits, no constraints (for example when it comes to energy, money and knowledge). You can do and become everything you ever wished for. Perhaps you would like to run your own business, write a successful book, build your own house or become a nomad traveling around the world. Think of what you want to do or become without any constraints. Again, everything is possible... you just have to let go of limitations that you have in mind. This might sound like a difficult exercise, but once you think of something you would really like to do or become, your thoughts will start to flow. Take at least five minutes and write down one or multiple things you wish, dream or hope to attain in the next 10 years. Please keep on writing (in English) and aim for a paragraph or two. Try to remember, everything is possible. If everything is possible, then in the upcoming years, I would like to. . ..”

# Questions to Your Future Self

| Tutorial | Experiment |
| --- | --- |
| 1. Future self, what did you have for breakfast? | 1. Future self, how are you feeling today? |
| 1. Future self, do you eat that every day? | 1. Future self, what are you celebrating? Who is coming? What are you guys going to do? |
| 1. Future self, where are you going on vacation this year? | 1. What does it feel like to finally achieve this? |
| 1. Future self, how much will this vacation cost? | 1. Future self, what did you do or change in the last years that helped you achieve this? |
|  | 1. What were some difficult experiences you overcame and how did you deal with them? |
|  | 1. What one lesson did you learn from these experiences? |
|  | 1. Future self, what advice would you give me to start working towards this goal? |
|  | 1. Future self, what are the best parts about being you? |
|  | 1. Think of a question you would like to ask your future self and ask it. Start the question with: Future self… |

# Means and Standard Deviations of Engagement, Embodiment and Perspective-Taking Questions

|  |  | *In Vivo*  (*N* = 30) | VR  (N = 31) |
| --- | --- | --- | --- |
| Embodied Perspective-Taking | When sitting in the chair of my future self, I felt as if I was my future self. | 5.37 (1.35) | 5.48 (1.23) |
|  | When sitting in the chair of my future self, I felt as if I was someone else. | 4.17 (1.74) | 3.74 (1.46) |
| Cognitive Perspective-Taking | When sitting in the chair of my future self, my thoughts changed from how I normally think. | 5.17 (1.15) | 4.58 (1.54) |
|  | I was surprised by the answers I came up with. | 4.67 (1.77) | 4.32 (1.56) |
|  | I gave different answers than I normally would have. | 4.37 (1.81) | 3.32 (1.54) |
|  | Perspective taking composite. | 4.73^*^ (1.76) | 4.08 (1.55) |

*Note:* ^*^ *p* < .05; ^**^ *p* < .01; ^***^ *p* < .001

# Descriptive Statistics and Correlations Between Conditions

*Means, standard deviations, and correlations with confidence intervals for the VR condition.*

| Variable | *M* | *SD* | 1 | 2 | 3 | 4 | 5 | 6 | 7 |
| --- | --- | --- | --- | --- | --- | --- | --- | --- | --- |
| 1. Connected T1 | 3.84 | 1.39 |  |  |  |  |  |  |  |
| 2. Similar T1 | 3.58 | 1.36 | .40* |  |  |  |  |  |  |
| 3. Liking T1 | 5.84 | 1.16 | .21 | .0 |  |  |  |  |  |
| 4. Vividness T1 | 4.23 | 1.48 | .52** | .34 | .58** |  |  |  |  |
| 5. Connected T2 | 4.90 | 1.49 | .59** | .44* | .28 | **.59**** |  |  |  |
| 6. Similar T2 | 4.13 | 1.41 | .47** | .53** | .24 | **.54**** | .63** |  |  |
| 7. Liking T2 | 6.32 | 0.60 | .26 | .21 | .32 | **.50**** | .45* | .46** |  |
| 8. Vividness T2 | 5.02 | 1.24 | .32 | .23 | .35 | **.75**** | .62** | .56** | .42* |

*Note.* *M* and *SD* are used to represent mean and standard deviation. T1 and T2 stand for baseline and post-experiment, respectively. The bolded correlations are referenced in the discussion* indicates *p* < .05. ** indicates *p* < .01.

*Means, standard deviations, and correlations with confidence intervals for the in vivo condition*

| Variable | *M* | *SD* | 1 | 2 | 3 | 4 | 5 | 6 | 7 |
| --- | --- | --- | --- | --- | --- | --- | --- | --- | --- |
| 1. Connected T1 | 3.57 | 1.79 |  |  |  |  |  |  |  |
| 2. Similar T1 | 3.23 | 1.43 | .30 |  |  |  |  |  |  |
| 3. Liking T1 | 5.50 | 1.04 | .08 | -.29 |  |  |  |  |  |
| 4. Vividness T1 | 3.43 | 1.63 | .62** | .21 | .23 |  |  |  |  |
| 5. Connected T2 | 4.13 | 1.43 | .41* | .29 | .30 | **.09** |  |  |  |
| 6. Similar T2 | 3.40 | 1.35 | .27 | .47** | .07 | **.19** | .47** |  |  |
| 7. Liking T2 | 6.23 | 1.10 | .42* | -.01 | .55** | **.40*** | .42* | .24 |  |
| 8. Vividness T2 | 4.62 | 1.27 | .36 | -.16 | .46* | **.41*** | .30 | .38* | .51** |

*Note.* *M* and *SD* are used to represent mean and standard deviation. T1 and T2 stand for baseline and post-experiment, respectively. The bolded correlations are referenced in the text. * indicates *p* < .05. ** indicates *p* < .01.
